# *De novo* assembly of a transcriptome from juvenile blue crabs (*Callinectes sapidus*) following exposure to surrogate Macondo crude oil

**DOI:** 10.1186/s12864-015-1739-2

**Published:** 2015-07-11

**Authors:** Bree K. Yednock, Timothy J. Sullivan, Joseph E. Neigel

**Affiliations:** South Slough National Estuarine Research Reserve, Charleston, OR USA; Department of Biology, University of Louisiana at Lafayette, Lafayette, LA USA

## Abstract

**Background:**

The blue crab, *Callinectes sapidus,* is economically and ecologically important in western Atlantic and Gulf of Mexico coastal estuaries. In 2010 blue crabs in the northern Gulf of Mexico were exposed to crude oil and chemical dispersants from the Deepwater Horizon oil spill. To characterize the blue crab transcriptome and identify genes that could be regulated in response to oil exposure we sequenced transcriptomes from hepatopancreas and gill tissues of juvenile blue crabs after exposing them to a water-accommodated fraction of surrogate Macondo crude oil in the laboratory and compared them to transcriptomes from an unexposed control group.

**Results:**

Illumina sequencing provided 42.5 million paired-end sequencing reads for the control group and 44.9 million paired-end reads for the treatment group. From these, 73,473 transcripts and 52,663 genes were assembled. Comparison of control and treatment transcriptomes revealed about 100 genes from each tissue type that were differentially expressed. However, a much larger number of transcripts, approximately 2000 from each tissue type, were differentially expressed. Several examples of alternatively spliced transcripts were verified by qPCR, some of which showed significantly different expression patterns. The combined transcriptome from all tissues and individuals was annotated to assign putative gene products to both major gene ontology categories as well as specific roles in responses to cold and heat, metabolism of xenobiotic compounds, defence, hypoxia, osmoregulation and ecdysis. Among the annotations for upregulated and alternatively-spliced genes were candidates for the metabolism of oil-derived compounds.

**Conclusions:**

Previously, few genomic resources were available for blue crabs or related brachyuran crabs. The transcriptome sequences reported here represent a major new resource for research on the biology of blue crabs. These sequences can be used for studies of differential gene expression or as a source of genetic markers. Genes identified and annotated in this study include candidates for responses of the blue crab to xenobiotic compounds, which could serve as biomarkers for oil exposure. Changes in gene expression also suggest other physiological changes that may occur as the result of exposure to oil.

**Electronic supplementary material:**

The online version of this article (doi:10.1186/s12864-015-1739-2) contains supplementary material, which is available to authorized users.

## Background

During the Deepwater Horizon oil spill (DHOS) in the summer of 2010, crude oil from the Macondo exploration oil well washed ashore along 1,600 km of coastline in the northern Gulf of Mexico [1]. Seven months after the spill, surface-soil concentrations of petroleum hydrocarbons were still as high as 590 mg g^−1^ in some coastal marshes [2]. The impact of the DHOS on coastal biota is still being determined, but negative effects on both resident [3] and transient [4] marsh species have been reported. The effects of the DHOS on the blue crab, *Callinectes sapidus*, is of particular concern because this species supports a nationally important commercial fishery and previous work has demonstrated detrimental effects of oil on the growth and survival of blue crabs [5, 6]. Large expanses of the marsh habitat used by blue crabs in the northern Gulf of Mexico were contaminated with oil [1] and acute reductions in blue crab recruitment in coastal marshes were observed following the DHOS [7]. Patterns of gene expression in a marsh fish (*Fundulus grandis*) at a location with only trace levels of oil from the DHOS were indicative of detrimental effects on physiology and reproduction [3].

Previous investigations of the effects of oil or major components of oil (*e.g.,* polycyclic aromatic hydrocarbons) on gene expression in crustaceans have been limited to a small number of known stress-response genes [8–11]. With the advent of high-throughput deep-sequencing methods such as RNA-Seq [12], transcriptome sequencing has become a practical alternative for discovering genome-wide changes in gene expression following exposure to xenobiotics and other forms of environmental stress. However, to date, transcriptomes have been generated for only a handful of brachyuran crab species [13–16]. This deficit is due in part to the challenges associated with assembling a transcriptome without a reference genome from the same or a closely-related species [17]. At present, the only published crustacean genome is from *Daphnia pulex* [18], a member of the class Branchiopoda, which is estimated to have diverged from the class Malacostraca, to which crabs belong, more than 400 mya [19].

This study used RNA-Seq to examine short-term transcriptomic responses in two tissues from juvenile blue crabs exposed to crude oil in a laboratory exposure experiment. Because quantities of oil from the Macondo oil well are limited, a widely-used surrogate oil that has similar chemical and toxicological properties and that is recommended by the Gulf of Mexico Research Initiative was used for our experiments. Juvenile blue crabs were used rather than adults so that exposures could be conducted in relatively small volumes (3 L) of water and because previous research on the sublethal effects of the water-soluble fraction of South Louisiana crude oil on juvenile blue crabs could be used to inform our experimental design. These earlier studies demonstrated sublethal effects in the range of 0.8 to 2.5 ppm [6, 20], therefore we used a concentration of 2.5 ppm to produce a short-term stress response that would not be lethal.

## Results

### Exposure experiment

Following the 24 h oil exposure, all of the juvenile crabs were alive. Dissolved oxygen levels were reduced in all exposure chambers from the initial readings of 5.9 mg l^−1^, with the treatment group showing the lowest final values (Table 1). Hypoxia in the Gulf of Mexico is often defined as dissolved oxygen levels below 2 mg l^−1^ [21] based on regional values for water temperature, salinity, and oxygen saturation potential. Using this definition, several crabs were experiencing hypoxic conditions by the end of the exposure experiment (Table 1). Ammonia levels increased in all chambers (Table 1), but never exceeded the range considered acceptable for aquaculture of blue crabs [22].

**Table 1:** Dissolved oxygen and ammonia levels before and after oil exposures

		Dissolved Oxygen (mg l^−1^)		Ammonia (ppm)	
Crab	CW (mm)	Start	End	Start	End
C1	15	5.9	4.13	0	<0.25
C2	33	5.9	2.11	0	0.5
C3	37	5.9	3.15	0	0.25 – 0.5
C4^a^	32	5.9	4.75	0	0.5
T1	17	5.9	4.27	0	<0.25
T2	28	5.9	1.6^b^	0	0.5
T3	44	5.9	0.55^b^	0	0.5
T4	31	5.9	1.45^b^	0	0.5

Levels of dissolved oxygen and ammonia in each exposure chamber at the start and end of the 24 h static oil exposure. C1-C4 = control group; T1-T4 = treatment/oil group

^a^Identified as *Callinectes similis* following the oil exposure

^b^Below the 2.0 mg l^−1^ threshold used to define hypoxia in the Gulf of Mexico [21]

### RNA quantity and quality

**Fig. 1:**
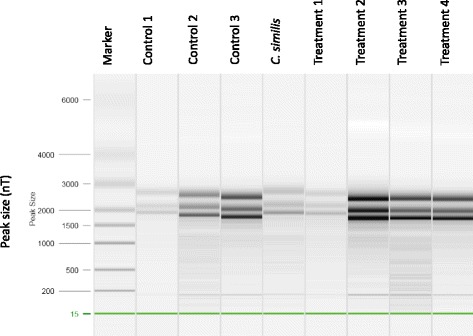
Unusual three band pattern of total RNA from juvenile crabs. QIAxcel System (Qiagen) gel image from the quality control assessment of total RNA samples extracted from hepatopancreas tissue used for cDNA library preparation and Illumina sequencing. RNA from gill tissue showed the same three band pattern

RNA extracted from hepatopancreas and gill tissues of the eight juvenile crabs used in the exposure experiment was of sufficient quantity and quality for cDNA library preparation. RNA yields from gill tissue (mean 1.49 μg, SD 1.0 μg) were consistently much lower than for hepatopancreas tissue (mean 21.0 μg, SD 11.6 μg). The electrophoretic pattern produced by all RNA samples on a QIAxcel System (Qiagen) showed a surprising three-band pattern, rather than the normal two bands corresponding to 18S and 28S ribosomal RNA (Fig. 1). Although rarely reported, this pattern has been documented for other crustaceans and is apparently due to the separation of the 28S ribosomal RNA into two smaller fragments [23]. Prior to cDNA library preparation, equal quantities of RNA from crabs in the respective control and treatment groups were pooled and run on a Bioanalyzer System (Agilent Technologies, Inc.) at the University of California Davis DNA Technologies Core Facility. The RNA was of high enough quality (RIN 4.9 – 5.5) for cDNA library preparation and Illumina sequencing. Three distinct ribosomal RNA peaks were resolved on the Bioanalyzer as well.

### Illumina sequencing

**Fig. 2:**
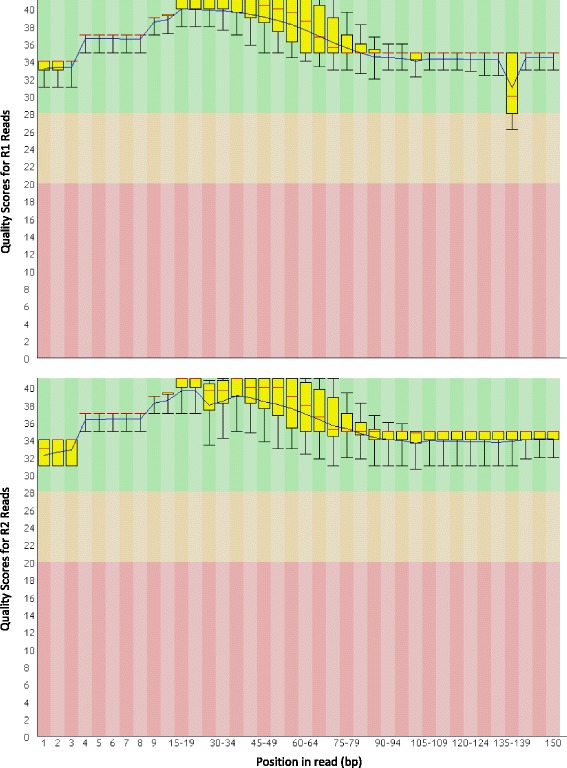
Per base quality scores for sequencing reads after quality filtering. Per base quality scores from the FastQC analysis of R1 (above) and R2 (below) sequencing reads from the control group’s gill tissue cDNA library after quality filtering and trimming. These figures are representative for all libraries showing high quality scores at all bases, with the exception of bases 135–140 in the R1 (above) sequences. This reduction in quality scores was seen for all four libraries and indicates an artefact of the sequencing run. Yellow boxes = 25 – 75 % interquartile range; whiskers = 10 – 90 %; red lines = median; blue line = mean

Over 174 million 150 bp raw sequencing reads were obtained from the combined cDNA libraries (Table 2). Gill libraries produced fewer reads than those from the hepatopancreas, but still totalled over 18.6 and 19.5 million paired-end reads for the control and treatment groups, respectively (Table 2). After quality filtering and trimming, 98 % of the reads from each library were retained, with read lengths ranging from 20–150 bp and high FastQC quality scores (>28) for most positions in sequencing reads from both directions (*i.e.,* R1 and R2). A drop in average FastQC quality scores was seen across positions 135–140 in R1 reads (Fig. 2), reflecting a high proportion of uncalled bases (*i.e.,* Ns). FastQC also identified nucleotide biases in the first 10 positions of the reads, which resulted in inflated per-base and per-sequence GC content. This pattern is consistent with a well-known priming bias of the random hexamer oligos used in TruSeq Illumina cDNA library preparation [24]. Sequence duplication levels were high, indicating good coverage of the transcriptome. No contaminant sequences were detected by FastQC. Of 314 highly-represented sequences identified by FastQC, 308 were mitochondrial sequences; the remaining six were 90 % identical to a trypsin-like serine proteinase mRNA reported from the related (family Portunidae) crab *Scylla paramamosain* (GenBank Accession: KC757380).

**Table 2:** Counts of paired-end reads per library before and after quality-filtering and trimming.

Experimental Group	Tissue	No. paired-end reads before trimming	No. trimmed paired-end reads
Control	Gill	18,671,390	18,331,683
Treatment	Gill	19,599,721	19,270,123
Control	Hepatopancreas	23,838,054	23,376,636
Treatment	Hepatopancreas	25,324,586	24,842,054

Number of paired-end sequencing reads from *C. sapidus* before and after quality trimming and filtering

### Transcriptome assembly and validation

The trimmed and quality-filtered sequences from all four libraries were used to assemble a transcriptome using the software package Trinity [25], run under BioLinux (version 7) on an HP Z800 workstation with 96 GB of RAM. A total of 73,473 transcripts (referred to as isoforms in the Trinity software package) were assembled and grouped across 52,663 genes (referred to as components in the Trinity software package). Contigs of the transcripts contained an average of 2,556 reads and averaged 1,183 bp in length. The N50 statistic was 2,377, which indicates at least 50 % of the sum of the lengths of all contigs include contigs that were at least 2,377 bp long. The N90 statistic (corresponding to at least 90 % of the sum of lengths) was 422.

**Fig. 3:**
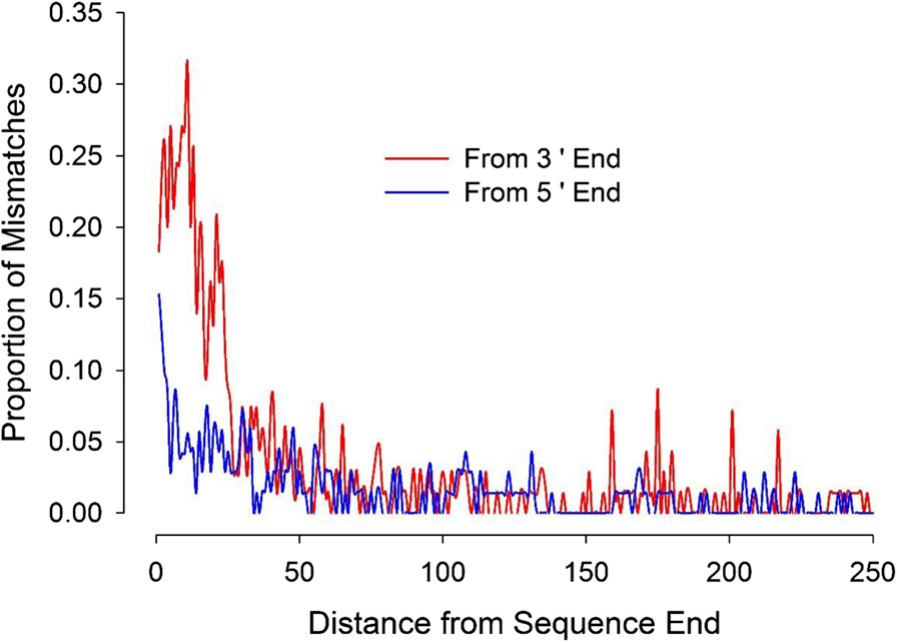
Position of bases in transcriptome sequences that are mismatched with published sequences. The proportion of bases mismatched between published *C. sapidus* sequences and the transcriptome versus distance from the end of the transcriptome sequence. Mismatches were counted for 87 Needleman-Wunsch alignments of various cDNA sequences retrieved from GenBank for *C. sapidus* with sequences retrieved by BLASTN searches of the transcriptome assembly reported here

The quality of the transcriptome assembly was tested by comparisons between known genomic sequences of blue crabs and the transcripts assembled by Trinity. Sequences from the assembled transcriptome were compared with partial exon sequences of five different protein-coding genes that had previously been determined for over 800 individual blue crabs in a population genetic study [26]. BLASTN searches returned a single high-scoring hit for each query, and each hit sequence aligned with the full length of the query sequence without indels or nucleotides that didn’t match known sequence variants (Table 3). This indicates that sequencing error rates were very low for these regions, although all were at least 241 bp from the 3’ end and 261 bp from the 5’ end of the assembled transcript where coverage would be lower. To assess sequencing error rates for regions closer to the ends of the assembled transcripts, additional alignments were made between blue crab cDNA sequences from the NCBI sequence database (GenBank) and sequences from our assembled transcriptome. BLASTN searches returned at least one high-scoring hit for 87 out of 100 cDNA sequences used as queries. In cases where multiple hits occurred for a single query the match with the longest alignment was selected. For each of these, the original full-length query cDNA was realigned with the transcriptome sequence using the Needleman-Wunsch algorithm to produce a global alignment [27]. In these alignments, the proportion of mismatches was highest at the ends of these alignments where there are typically lower numbers of sequencing reads (Fig. 3).

**Table 3:** BLAST results from the transcriptome validation with previously published DNA sequences from blue crabs

Locus	Query Length	Start of Query in Alignment	Alignment Length	Number of Polymorphic Sites
*ant*	414	505	1355	35
*atps*	227	316	802	37
*hsp*	489	1428	2238	28
*rpl*	191	241	786	11
*tps*	368	1902	3368	19

Summary of BLASTN searches of the transcriptome using published sequences from five protein-coding genes as queries. No unknown polymorphic sites were found in the transcriptome sequences

### Transcriptome annotation

**Fig. 4:**
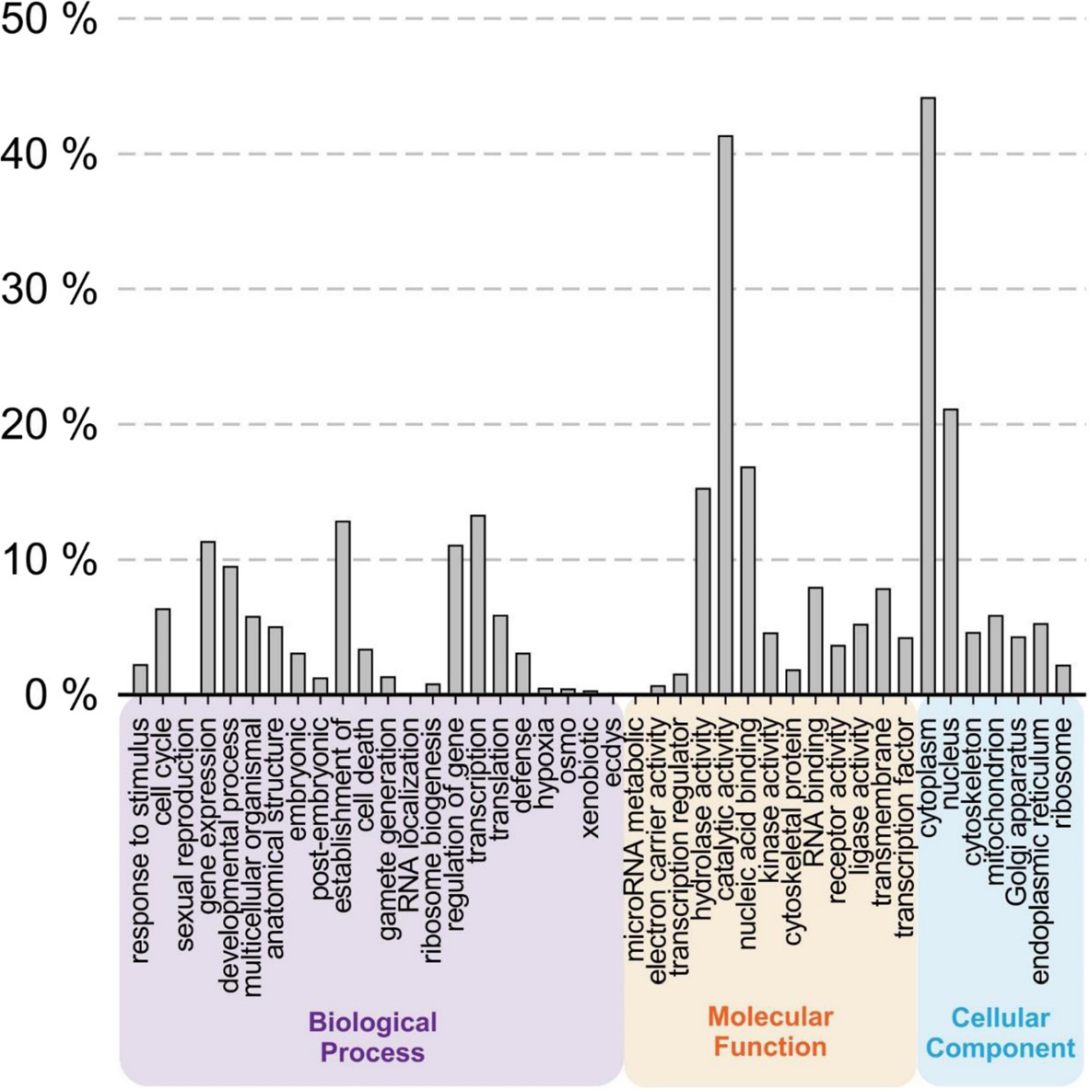
Gene Ontology (GO) term distribution of BLASTX hits for the combined transcriptome of *Callinectes sapidus.* The longest transcript of each gene sequence was blasted against the SwissProt protein sequence database. GO categories were chosen to match those used by Zeng *et al.* [17] for annotation of the transcriptome of the crustacean *Parhyale hawaiensis*

Of 52,663 putative gene sequences (Trinity components) used as queries in BLASTX searches of the SwissProt protein sequence database, there were significant hits for 32,757 (62 %). For the database sequences with the highest bitscores in each search, 31,416 had at least one associated GO term with an average of 16.6 GO terms each. A total of 19,635 unique GO terms were identified, and a total of 149 unique GO terms from the GO Consortium Generic slim ontology. To allow a comparison of the GO term distribution of the transcriptomes of *C. sapidus* with those shown by Zeng *et al.* [17] for several other arthropods including a novel transcriptome for the marine amphipod *Parhyale hawaiensis*, we used the groupings of GO terms shown in their Fig. 3. The distribution of GO terms for the *C. sapidus* transcriptome appears to be more similar to those for the other crustaceans: *P. hawaiensis* and *Daphnia pulex* than for that of the insect, *Drosophila melanogaster*, but also similar to the insect *Oncopeltus fasciatus* (Fig. 4; see Zeng *et al.* [17] Fig. 3).

SwissProt sequences found by the BLASTX searches described above that had GO annotations (including those derived from “is-a” relationships) that contained words that matched any of the following terms of interest: ‘cold’, ‘defense’, ‘detox’, ‘ecdys’, ‘heat’, ‘hypoxia’, ‘osmo’ and ‘xenobio’ are listed in Additional file 1. Some of the sequences thus identified appear to have close functional relationships to the selected term, while others that have broader functions are presumed to be involved indirectly. For example the term ‘xenobio’ matched annotations for cytochrome P450’s and UDP-glucuronosyltransferases, which are known components of Phase I and Phase II metabolism for xenobiotic compounds in blue crabs [28], but ‘xenobio’ also matched annotations for cyclin-dependent kinase 9, which has a more general role in cell cycle regulation. There were a large number (998) of sequences identified with GO annotations that matched the term ‘defense’. Many of these could be placed into one of five broad categories: 1) cationic antimicrobial peptides such as beta-defensin 1, 4 and 17, drosocin, uperin, and lycotoxin; 2) pattern recognition proteins including C type lectin domain family 2 and 4, Toll-like receptor 2, 3, 11, and NACHT, LRR and PYD domains-containing protein 8; 3) proteins involved in ubiquitination including peroxiredoxin, NADH dehydrogenase, ubiquitin conjugating enzyme, ubiquitin-40S ribosomal protein S27a, E3 ubiquitin-protein ligase AMFR, TRIM21, RNF103; 4) mucin and mucin 4 genes; and 5) inflammation including nitric oxide synthase and interleukin-1, 2, 11, 12, and 34. As was the case for sequences with GO terms that matched ‘xenobio’, many of the sequences with annotations that matched ‘defense’ did have obvious direct roles in defence functions, but could have indirect roles through mediation of signal transduction, regulation of transcription or other generalized functions.

### Differential expression and annotation

**Fig. 5:**
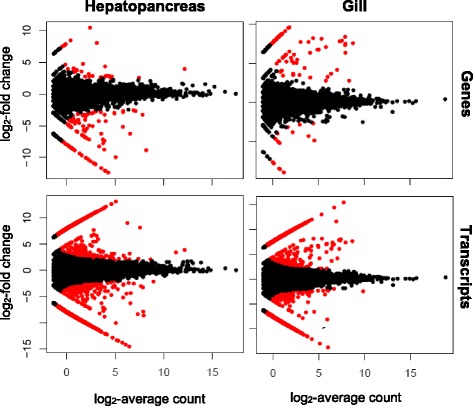
Differential expression of genes and transcripts across treatments. Log_2_-fold change between control and treatment group (y-axis) plotted against the log_2_-average expression of both groups (x-axis). Each point represents one gene or transcript. Red indicates genes and transcripts that are significantly differentially expressed in each tissue at a false discovery rate of 0.05. Black points are not significantly differentially expressed

Differential expression in response to oil exposure was examined for both the gill and hepatopancreas cDNA libraries using edgeR [29]. Differential expression of transcripts was based on the 73,473 sequences assembled and characterized by Trinity as *isoforms*, while differential expression of genes was based on the 52,663 sets of sequences that Trinity grouped into *components*. Thus we are assuming that Trinity’s components correspond to genes and that isoforms belonging to the same component correspond to alternatively-spliced transcripts of the same gene. A total of 2,424 and 2,136 transcripts from the hepatopancreas and gill libraries, respectively, showed significant differential expression between the control and treatment groups at a false discovery rate of 0.05 (Fig. 5). Changes of expression levels for upregulated transcripts ranged from 2.1 to 15.5 log_2_-fold change in gill tissue and 2.0 to 13.2 log_2_-fold change in hepatopancreas tissue. Downregulated transcripts showed log_2_-fold change decreases in expression ranging from −2.0 to −13.8 in gills and −2.0 to −14.5 in hepatopancreas.

The number of genes that were differentially expressed was much lower than the number of transcripts for both gill (90) and hepatopancreas (136) cDNA libraries (Fig. 5). This is to be expected due to the clustering of multiple transcripts into each Trinity component. Of the 136 genes that showed significant differential expression levels in hepatopancreas tissue, 104 were down-regulated (−2.5 to −12.4 log_2_-fold change) and 32 were upregulated (2.6 to 10.5 log_2_-fold change). Changes in the expression levels of genes in the gill ranged from log_2_-fold change of −2.7 to −8.9 for downregulated genes and 2.8 to 9.6 for upregulated genes.

Genes and transcripts with the largest differences in representation between oil exposure and control libraries were annotated with Blast2GO. Subsets of these annotations for hepatopancreas libraries are shown in Tables 4 and 5, additional annotations for both hepatopancreas and gill libraries are provided in Additional file 2. Although the functional significance of up or downregulation of these genes in response to oil exposure has not been determined, some of the annotations suggest possible functions for upregulated genes and transcripts in xenobiotic metabolism.

**Table 4:** Annotated genes with largest differences in representation between oil treatment and control hepatopancreas libraries

Trinity Component ID	Blast2GO Annotation	Log_2_ Fold Change
comp22445_c3	Serine Threonine-Protein Kinase N-Like	10.5
comp16962_c2	Thiopurine S-Methyltransferase	8.5
comp9165_c1	NADH Dehydrogenase Subunit 4	8.1
comp28129_c0	Nuclease Harbi1-Like	7.9
comp6688_c0	Elastin A	7.9
comp15366_c0	Probable C-5 Sterol Desaturase 1-Like	7.7
comp10045_c0	D-3-Phosphoglycerate Dehydrogenase	7.7
comp8161_c0	Regulating Synaptic Membrane Exocytosis Protein 2-Like	7.5
comp26975_c0	NADH Dehydrogenase Subunit 4	7.2
comp5295_c0	Leukocyte Receptor Cluster Member	4.1
comp16233_c0	Cuticle Proprotein	−12.4
comp8677_c0	Calcified Cuticle Protein	−11.7
comp14403_c0	Gastrolith Protein 10	−11.1
comp8839_c0	Tyrosine Recombinase	−10.1
comp23500_c1	Carboxylesterase	−8.8
comp7612_c0	Cuticular Protein Analogous To Peritrophins 3-A1 Precursor	−8.6
comp23080_c0	Fibrocystin-L-Like	−8.1
comp13004_c0	Leucine-Rich Repeat-Containing Protein DDB_G0290503-Like	−7.8
comp13665_c0	Cuticle Protein BD1	−7.7
comp9338_c0	Crustin Partial	−7.5

**Table 5:** Annotated transcripts with largest differences in representation between oil treatment and control hepatopancreas libraries

Trinity Isoform ID	Blast2GO Annotation	Log_2_ Fold Change
comp25500_c1_seq2	Dehydrogenase Reductase SDR Family Member 7	13.2
comp16829_c0_seq1	2-Hydroxyacylsphingosine 1-Beta-Galactosyltransferase-Like	12.7
comp26374_c0_seq3	Lysosomal Alpha-Mannosidase	12.7
comp26147_c0_seq4	AMP Dependent CoA Ligase	12.0
comp10045_c0_seq2	D-3-Phosphoglycerate Dehydrogenase	11.9
comp25478_c1_seq7	Hemocyanin Subunit Partial	11.9
comp26226_c0_seq1	ATP-Dependent RNA Helicase DHX33	11.6
comp22020_c0_seq1	Transmembrane Protein 45B	11.4
comp24411_c0_seq3	Paxillin-Like Isoform X1	11.1
comp26306_c0_seq2	CCA tRNA Nucleotidyltransferase Mitochondrial	10.9
comp24732_c0_seq2	Receptor Expression-Enhancing Protein 5-Like	−14.6
comp4958_c0_seq1	Cytoplasmic Manganese Superoxide Dismutase	−14.2
comp20628_c0_seq1	Cuticle Proprotein	−13.8
comp23016_c0_seq2	Beta-1,3-Galactosyltransferase 6	−13.4
comp16538_c0_seq2	Group XV Phospholipase A2-Like	−13.3
comp25764_c0_seq1	Peptidyl-Prolyl Cis-Trans Isomerase SDCCAG10	−12.8
comp16233_c0_seq1	Cuticle Proprotein	−12.3
comp8677_c0_seq1	Calcified Cuticle Protein	−11.7
comp23980_c0_seq2	Nascent Polypeptide-Associated Complex Subunit Muscle-Specific Form-Like	−11.6
comp23010_c0_seq3	Creatininase	−11.4

The transcripts with the largest differences in expression between the gill libraries of the control and treatment groups were myosin and tropomyosin (Additional file 2). The products of both of these genes are structural components of muscle tissue, therefore we suspected that some muscle tissue attached to the gills was inadvertently retained during dissection and included in the RNA extraction. We validated these results using qPCR: the two smallest crabs in the treatment group had relatively high levels of myosin and tropomyosin expression, while the other individuals showed no expression of these transcripts. Because of this apparent tissue heterogeneity in gill libraries, discussion of our differential expression results focuses on the results from hepatopancreas libraries.

Often was the case that genes showing significant differences in expression between the two groups being compared was the result of only one transcript or a small number of transcripts being upregulated. For example, Trinity component comp10045_c0 corresponds to the gene that encodes D-3-phosphoglycerate dehydrogenase, which was found to be significantly upregulated (4.4 log_2_-fold change, FDR = 1.21 × 10^−05^). However, only one of the two transcripts associated with this component (comp10045_c0_seq2) was significantly upregulated (11.9 log_2_-fold change, FDR = 9.78 × 10^−13^).

In many cases some transcripts of a gene were downregulated while other transcripts of the same gene were upregulated, resulting in no net difference of gene expression. For example, three transcripts for a hemocyanin subunit (comp25478_c1_seq7, comp25478_c1_seq17, comp25478_c1_seq10) were highly differentially expressed (7.9 to 11.9 log_2_-fold change) in the hepatopancreas, but the remaining 18 hemocyanin subunit transcripts and the corresponding gene to which all of these transcripts clustered (comp25478_c1; GenBank Accession: AF249297.1) were not.

Differential expression patterns were not the same for both tissues (*i.e.,* genes and transcripts that were differentially expressed in one tissue following oil exposure were not differently expressed in the other tissue). To characterize transcriptome profiles for the different tissues, comparisons were made between the gill and hepatopancreas libraries of the control group. As would be expected, a much larger number of transcripts (10,955) were differentially expressed between tissue types. Unlike the gill library from the treatment group, which appears to represent mixed tissue types, there were no transcript or gene expression patterns that indicate the presence of mixed tissue in the gill library from the control group. Therefore this cross-tissue comparison of expression patterns provides valuable insight into genes that may have tissue-specific functions.

### Validation of expression levels using qPCR

**Fig. 6:**
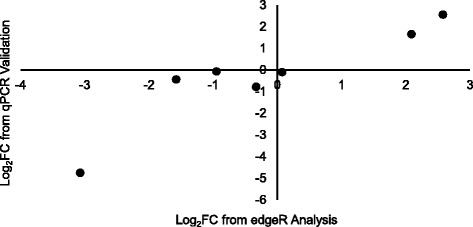
Validation of edgeR differential expression results with qPCR. Log_2_-fold changes from the edgeR differential expression analysis (x-axis) were highly correlated with log_2_-fold change values from quantitative real-time PCR (qPCR; y-axis). Log_2_-fold changes are relative to the control group. The seven data points represent three genes (*hsp90*, *egl9*, and *hemocyanin*) and four transcripts (*rpl*, *got2*, *sdr1*, *ugt1*)

Quantitative real-time PCR (qPCR) was used to measure the expression levels of three genes, four transcripts, and two alternatively-spliced transcript variants to evaluate the accuracy of the differential expression patterns characterized by the analyses done using the edgeR software package [29] and described in the above section. The genes, transcripts, and variants that were selected for qPCR validation (Table 6) include a range of expression levels and targeted functional annotations (from Blast2GO) that indicate potential roles in xenobiotic metabolism. Overall, expression patterns between the control and treatment groups for both methods (*i.e.,* edgeR and qPCR) were highly correlated (r = 0.9169, *p* = 0.0037; Fig. 6). However, there was variation in expression levels among individuals. For example, the log_2_-fold change in expression levels of *egl9* and *hemocyanin* were positive for treatment individuals T2, T3, and T4, but negative in treatment individual T1 (Table 7). The enzyme encoded by *egl9* is known to be an important regulator in hypoxic response (reviewed in [30]), therefore elevated expression of this gene may be attributed to the mildly hypoxic conditions experienced by individuals T2, T3, and T4 (Table 1). Treatment individual T1 did not experience hypoxic conditions (Table 1) and showed no upregulation of hypoxia-associated transcripts (Table 7).

**Table 6:** Description of primers used for qPCR validation of differential expression results

Gene (abbreviation)	Trinity Component or Isoform ID	Primer Name	Sequence (5’ to 3’)	Product Size (bp)
Heat Shock Protein 90 (*hsp90*)	comp22653_c0^a^	HSP90F2	CACCGACAACATCAAGCTGTAC	93
		HSP90R2	ACACCACGCACAAAGTTGAG	
EGL9 (*egl9*)	comp25287_c2^a^	EGL9F1	TGTTTTCGGCCTAAGTGGTG	111
		EGL9R1	AAATCCCGCACTATGCTTGG	
Hemocyanin	comp16095_c0^a^	HEMF1	TAACAAGAACCCGGGCAAAG	71
		HEMR1	AAATGAAGGTCCTGCTGGTG	
Ribosomal Protein L12 (*rpl*)	comp18523_c0_seq1	RPNF2	AATCGCAGTTCATCCTCCAC	71
		RPNR2	GAGGCATGGTGCTGAATTTG	
Glutamate Oxyacetyl Transaminase 2 (*got2*)	comp4115_c0_seq1	GOTF2	TTGAGATGATGGGCACTCAG	85
		GOTR2	TCTTTCAGCATCAGCACCAG	
Short-chain Dehydrogenase Reductase (*sdr1*)	comp25500_c1_seq1	SDRF1	GTGCCGTAGTCATTATTCTCAGC	78
		SDRR1	TGTGGTGGTTTGACATGCAC	
*sdr1*-alternatively spliced variant (*sdr1*-ASV)	comp25500_c1_seq2	SDRF3	CTTTCACTGCGACAAAAATCGG	136
		SDRR3	CCTTGTTAACGAACCACCACTG	
Glucuronosyltransferase (*ugt1*)	comp16829_c0_seq2	UGTF2	AGCCAAGCAGGATGAGACTAAG	76
		UGTR2	TCTTGCATCAACACCAACGC	
*ugt1*-alternatively spliced variant (*ugt1-ASV*)	comp19629_c0_seq1	UGTF3	CAGTGGATGTGCAAATAATCGTC	116
		UGTR3	TGGGGCATCAAAGTACTGGTAG	

^a^Component consists of multiple transcripts that are all amplified by the primer pair

**Table 7:** qPCR results for treatment and control individuals

Gene ID	Trinity Isoform ID	Individual	Relative Quantitation (Log_2_)
*rpl*	comp18523_c0_seq1	C1	−0.539
		C2	−0.150
		C3	0.806
		T1	0.030
		T2	−0.037
		T3	0.363
		T4	−0.202
*got2*	comp4115_c0_seq1	C1	0.619
		C2	0.294
		C3	−0.796
		T1	0.075
		T2	0.142
		T3	−0.258
		T4	0.306
*ugt1*	comp16829_c0_seq2	C1	−2.591
		C2	3.790
		C3	0.874
		T1	−0.211
		T2	1.367
		T3	1.516
		T4	2.573
*sdr1*	comp25500_c1_seq1	C1	−1.304
		C2	2.329
		C3	−0.151
		T1	−0.068
		T2	0.730
		T3	1.834
		T4	−4.589
*hsp90*	comp22653_c0_seq_1 & 2	C1	−4.509
		C2	3.421
		C3	3.737
		T1	−0.878
		T2	−2.614
		T3	−1.886
		T4	−2.239
*egl9*	comp25287_c2_seq_1, 2, & 3	C1	−4.053
		C2	2.790
		C3	3.581
		T1	−1.034
		T2	3.922
		T3	5.808
		T4	2.946
*hemocyanin*	comp16095_c0_seq1 & 2	C1	7.418
		C2	−0.337
		C3	−1.645
		T1	−5.403
		T2	9.374
		T3	9.420
		T4	1.106

Relative quantitation values for control and treatment individuals for validated genes and transcripts. C1-C3 = control group; T1-T4 = treatment/oil group

The alternatively-spliced variants of *sdr1* (*sdr1*-ASV) and *ugt1* (*ugt1*-ASV) were each only detected in single individuals. The *sdr1*-ASV was identified from treatment individual T3 (Mean Ct = 23.4), while the *ugt1*-ASV was identified from treatment individual T4 (Mean Ct = 23.5). Variation in expression across individuals, such as shown here, cannot be detected in RNA-Seq experiments with pooled individuals, and demonstrates the importance of validating patterns of gene expression in biological replicates.

### SNP discovery

The samtools package [31] identified a total of 357,034 single nucleotide polymorphisms (SNPs) in the transcriptome alignment file. This file includes alignment contigs for each of the 73,473 assembled transcripts and all of the sequencing reads that align to each transcript, therefore it encompasses all of the genetic variation in the pooled sample. It should be noted that homologous SNPs that appear in different transcripts could be counted multiple times. To validate a subset of these SNPs, we analysed the variable positions in the five contigs of the alignment file that correspond to the partial protein-coding sequences published in a comprehensive population genetic study − the same set of sequences used for the transcriptome assembly validation described earlier [26]. While it is not expected that a transcriptome generated from seven individuals would show all of the polymorphisms identified in the published sequence data set (generated from over 800 individuals), it is possible to use the sequences to evaluate whether or not the SNPs that were identified by samtools are real polymorphisms, rather than sequencing errors or assembly artefacts. The contig corresponding to one gene (*tps*) did not have any polymorphisms in the region that overlaps with the published sequences. For the remaining four genes in the data set, all of the SNPs that were identified by samtools in the regions of the contigs that correspond to the published sequences were confirmed. Many more SNPs were identified for each contig outside of the overlapping region (*i.e.,* located in sequence positions prior to the start of or after the end of the published sequences), but these could not be validated using these methods. For the contig corresponding to the gene that encodes ribosomal protein L12 (*rpl*, comp18523_c0_seq1), a tri-allelic SNP in the published data set was found in the assembled transcriptome alignment file after visual inspection of the contig alignment in the Tablet viewer [32], but it was not detected by samtools. This uncalled SNP is a result of the variant-calling algorithm used by samtools, which assumes a diploid individual with only two possible alleles. This limitation of the samtools package makes it inappropriate for estimating allele frequencies of pooled samples [33]. However, for the purpose of this study, which is simply to identify SNPs across the blue crab genome, this characteristic of the package will only make our results more conservative by limiting the number or SNPs to bi-allelic variants with relatively high frequencies in the sequenced sample.

## Discussion

This study reports the first transcriptomes for the blue crab, *Callinectes sapidus*. Prior to the publication of these transcriptomes, publically available sequence data for blue crabs were limited to 1,677 DNA and RNA sequences (many of which are from mitochondrial genes) and 10,930 expressed sequence tags (ESTs). The assembled and partially annotated transcriptomes reported here provide a valuable resource for transcriptomic and genomic projects on blue crabs and related taxa, and it offers over 300,000 potential SNPs that can be used for gene mapping and population genetic studies of blue crabs.

The oil exposure experiment identified hundreds of genes and thousands of transcripts that were significantly differentially expressed between the control and treatment groups, but it also highlights some important considerations for projects intended to measure the transcriptomic response of an organism to a specific treatment. First, this study used pooled RNA samples from multiple individuals in both treatment and control groups to characterize the response to oil. However, our qPCR validation assays of cDNA libraries from individual crabs revealed considerable variation among individuals within groups, both in which transcripts were differentially expressed and in levels of expression. This demonstrates the desirability of true biological replicates (*i.e.,* transcriptomes sequenced for each individual) to distinguish actual responses to a treatment from individual variation. However, despite this limitation, we identified novel candidates for the blue crab’s response to oil exposure. For example, the annotation for the gene with the second highest increase in transcript abundance (log_2_-fold change of 8.5) in the hepatopancreas following oil exposure was thiopurine S-methyltransferase (Table 4). In humans, thiopurine S-methyltransferase is known to catalyze the S-methylation of thiopurine compounds, including pharmaceutical compounds [34]. The annotation for the transcript with the greatest increase in abundance (log_2_-fold change of 13.2) in the hepatopancreas following oil exposure was dehydrogenase reductase SDR family member 7 (Table 5). This annotation also suggests a possible function in the response to oil exposure; in humans products of the SDR family of genes are involved in metabolic pathways including biotransformation of xenobiotic compounds [35]. These candidates suggest new gene products and pathways to investigate for the metabolism of oil-derived compounds and other xenobiotics. The transcriptomes from this study also can provide sequences for genes known from prior work to be important in xenobiotic metabolism. For example, BLAST searches of the blue crab transcriptome identified sequences for cytochrome P-450 products (CYP gene family) and glutathione-S-transferase (GST family), enzymes known to be important in the metabolism of polycyclic aromatic hydrocarbons by blue crabs (reviewed by [28]).

The second important consideration we note is problems that arise from variation in transcriptomes across tissues. When whole organisms are used in a gene expression study, as is often done with larvae or microscopic organisms (*e.g.,* copepods, amphipods, *Daphnia*), it is impossible to resolve the transcriptomes from distinct tissues after they have been combined. We were surprised to find that excision of a discrete and easily recognizable structure (gill) from small individuals did not yield a transcriptomically consistent set of tissues. From these considerations it can be concluded that patterns of gene expression must be carefully interpreted to avoid confounding treatment effects from other sources of transcriptomic variation.

We identified and validated several examples of alternatively-spliced transcripts in the blue crab transcriptome. Alternative splicing of pre-mRNA can significantly increase the complexity of transcriptomes and proteomes by producing multiple mRNA transcripts from a single coding sequence [36]. This phenomenon has been well studied in model organisms and is known to be quite common for a number of genes in humans [37]. Direct evidence of alternative splicing has also been reported for crustaceans [38], but scarcely mentioned in studies of decapod transcriptomes (*e.g.,* [39, 40]). Genome-wide inhibition of pre-mRNA splicing, has been observed as a part of the cellular response to stress in model systems [41]. The translation of genes that are not immediately needed for the stress response is repressed, while genes involved in protein folding and other stress-related functions are spliced normally [42, 43]. Our results suggest that differential splicing could also be important in the stress response of blue crabs. Studies that focus on changes in expression at the level of the gene could miss potentially important changes in expression resulting from alternative splicing. Future investigations into functional differences of alternatively spliced transcripts in blue crabs will undoubtedly improve our understanding of these mechanisms.

## Conclusions

This study has provided the first large-scale transcriptomic resource for the blue crab and the genus *Callinectes* as a whole, which will provide researchers with critical sequence data to target candidate genes or scan large genomic regions for molecular ecological, biochemical, and population genetic research. We also have identified genes and transcripts that are likely to play a role in responding to oil exposure. These will be important starting points for furthering our understanding of responses to oil exposure and other forms of stress from physiological, molecular, and biochemical perspectives. Lastly, we have identified a potential role for widespread differential splicing of pre-mRNA in the response to oil exposure. The possibility that this represents a generalized mechanism to prevent translation of genes not immediately involved in stress responses should be investigated.

## Methods

### Ethics statement

This experiment was conducted in accordance with policies established by the University of Louisiana at Lafayette Institutional Animal Care and Use Committee.

### Crab collection and WAF preparation

Eight small juvenile crabs (15–44 mm CW) were collected with dip nets during flood tide from a salt marsh creek in Cocodrie, Louisiana, in May of 2011. The crabs were acclimated for 16 h in individual 3 L glass exposure chambers containing filtered 20 ppt salinity seawater with aeration. The exposure chambers were kept in a 24 °C water bath during acclimation with 7 h of light followed by 9 h of darkness. A water-accommodated fraction (WAF) containing 30 ppm surrogate oil was prepared following the methods outlined in Singer *et al.* [44]. The WAF was prepared in a 2 L glass aspirator bottle by adding 45 μl of surrogate oil to 1.5 L of filtered 20 ppt salinity seawater and stirred with a magnetic 5 cm hexagonal stir bar for 24 h. Stirring speed during the 24 h period was maintained at the highest speed possible where no vortex developed. A control no oil “WAF” was prepared in the same way, but without the addition of oil, and both were allowed to settle for 45 min after mixing prior to use.

### Oil exposure

After the 16 h acclimation period, 95 % of the water from each chamber was removed and 2 L of filtered seawater (20 ppt salinity) was added. For the treatment group, 0.25 L of the concentrated 30 ppm WAF was transferred to each treatment chamber through silicone tubing from the bottom of the aspirator. For the control group, 0.25 L of the control no oil “WAF” was transferred to the control chambers in the same manner. All chambers were then slowly filled to a complete volume of 3 L with 20 ppt filtered seawater, creating a final WAF concentration of 2.5 ppm for the treatment group. Dissolved oxygen and ammonia levels of the water in the exposure chambers were 5.9 mg l^−1^ and 0 ppm, respectively. All eight exposure chambers were sealed with glass lids and placed in the 24 °C water bath for a 24 h period (12 h darkness followed by 12 h light).

### RNA extraction, library preparation, and Illumina sequencing

Following the 24 h static exposure, the water from each jar was gently decanted and each crab was carefully removed. The gills and midgut glands (hepatopancreas) were removed from each crab using sterile, RNase-free metal forceps and separately placed in 1.5 ml microcentrifuge tubes containing 1000 μl of chilled RNA*later®* (Ambion, Inc.). Tubes were kept at 4 °C or on ice until the RNA extraction.

After careful examination of the specimens under a microscope during the tissue dissections, it was determined that one of the juvenile crabs in the control group was not *C. sapidus*, but its congener *C. similis* (species identity was further confirmed with DNA sequences from a portion of the 16S ribosomal RNA gene). Consequently, RNA was extracted from this specimen and separate cDNA libraries were created for this individual for another study. This resulted in the control group being reduced to three individuals. All four crabs in the treatment group were confirmed to be *C. sapidus*.

RNA was extracted separately from each tissue sample using the Qiagen RNeasy Mini kit and protocol. RNA quantity of each sample was measured on a NanoDrop1000 (ThermoScientific) and quality was assessed using a QIAxcel System (Qiagen). All RNA samples were then stored at −80 °C. Prior to preparing the cDNA libraries for Illumina sequencing, two separate pools of RNA were made for the treatment group by combining equal quantities of total RNA extracted from the gill tissue of the four crabs into one pool and RNA extracted from the hepatopancreas in the second pool. The same was done for the three crabs in the control group. All four RNA pools were stored at −80 °C until they could be shipped overnight on dry ice to the University of California Davis DNA Technologies Core Facility where the cDNA libraries were prepared using the Illumina’s TruSeq cDNA kit. Paired-end sequencing of 150 bp reads was done for six cDNA libraries (4 *C. sapidus* and 2 *C. similis*) in one lane on an Illumina HiSeq2500 high throughput sequencer.

### Transcriptome assembly and validation

Quality control of the raw sequencing reads was done for each library using the program FastQC [45]. This program provides summary statistics for read quality, length, and GC content, and checks for over-represented and potential contaminant sequences. Low quality reads were removed or trimmed from each library using the program sickle [46]. Sequence data from each of the libraries was analysed a second time with FastQC after the quality-trimming step to confirm improvement in quality scores. The quality-filtered paired-end reads from all four libraries were concatenated into two separate files based on read direction then used to assemble a transcriptome using the program Trinity (version Trinity_r2013_8-14) [47]. The assembly was done on a Linux Desktop Workstation (HP Z800, Xeon E5530 processor with 96 GB of RAM) with the following parameters: Trinity.pl --seqType fq --JM 40G --left all_R1_reads.fastq --right all_R2_reads.fastq --CPU 6.

Sequences from the assembled transcriptome were compared with partial exon sequences of five different protein-coding genes that had previously been determined for over 800 individual blue crabs in a population genetic study [26]. To assess sequencing error rates for regions closer to the ends of the assembled transcripts, additional alignments were made between blue crab cDNA sequences from the NCBI sequence database (GenBank) and sequences from our assembled transcriptome.

### Annotation of transcriptome components

For annotation of the entire transcriptome, the following pipeline (written in Perl) was used. First, from the set of all sequences assembled by Trinity, a fasta file with one sequence for each gene (Trinity component or subcomponent) was created. For components represented by multiple isoforms, the longest sequence was used. If there was a tie for longest, the sequence with the lowest Trinity sequence number was used. Each of these representative gene sequences was then used as a query in a BLASTX search of the SwissProt subsection of the UniProt protein sequence database (Release 2015-04-25). If one or more significant hits were found in the database, the sequence with the highest bitscore was used as the basis for annotation. The Gene Ontology (GO) id’s associated with this sequence were then retrieved from the UniProt gene association database (Release 2015-04-25). All GO terms corresponding to these GO id’s were then retrieved from the GO database (Release 2015-05-16) and stored as a list. GO terms with “is-a” relationships to those listed in the gene association database were added to this list. This list of GO terms was then searched for matches with regular expressions corresponding the GO categories used in Zeng *et al.* [17] as well as the following terms of interest: ‘cold’, ‘defense’, ‘detox’, ‘ecdys’, ‘heat’, ‘hypoxia’, ‘osmo’ and ‘xenobio’. The number of genes matching each of these categories was then counted and lists of sequences with associated GO terms were generated (Additional file 1).

### Transcript abundance and differential expression

Transcript abundance for each library was accomplished in two steps. First, the quality-trimmed sequencing reads of the library were mapped to the assembled transcriptome using the Bowtie assembler program [48]. Next, the R package RSEM [49] was used to characterize expression levels for each transcript by estimating the abundance of sequencing reads that aligned to the transcript. The Trinity assembler clusters all transcripts and alternatively-spliced variants of the same gene into components, therefore expression levels were also estimated for every gene in the transcriptome by estimating the abundance of all of the sequencing reads that mapped to all of the transcripts within a Trinity component in the assembly. Differential expression was then tested by comparing expression across libraries for genes and individual transcripts using the R package edgeR [29]. The significance of differential expression was evaluated at false discovery rate of 0.05.

### Annotation of differentially expressed genes and transcripts

Blast2GO (version 3.0) was used to annotate both differentially represented genes and transcripts. Annotations were based on weighted and combined results of BLASTX searches of the NCBI non-redundant database and InterProScan searches for signatures of protein families and domains. Default parameters for Blast2GO were used for all stages of the annotation process.

### Validation of differential expression using qPCR

Quantitative real-time PCR (qPCR) was used to validate transcriptome expression levels for each library by measuring the transcript and gene expression levels separately for each crab used in the exposure experiment. Tissue-specific RNA from each juvenile crab was reverse transcribed into first strand cDNA using a High-Capacity cDNA Reverse Transcription Kit (Life Technologies). Primers were designed using the qPCR settings of Primer3 [50] and optimized for qPCR on an ABI StepOnePlus Real-Time PCR platform (Life Technologies) thermocycler. All primer sequences and product sizes are shown in Table 6. The total volume for qPCR reactions was 15 μl, consisting of 1.5 μl (10X) AmpliTaq Gold® PCR Buffer (Applied Biosystems), 1.5 μl (25 mM) MgCl_2_, 1.2 μl (10 mM) dNTPs, 0.9 μl (20 μM) of each forward and reverse primer, 0.6 units of AmpliTaq® Gold (Applied Biosystems), 0.15 μl 10X SYBR® Green 1 Nucleic Acid Gel Stain (Invitrogen), 5 ng of cDNA, and milliQ water. All qPCR reactions were run in triplicate with the following profile: 95 °C for 10 min, followed by 40 cycles of 15 s at 95 °C and 1 min at 60 °C, and ended with a melt curve from 60 °C to 95 °C. For direct comparison with the results of the edgeR analysis, qPCR results (relative quantitation) were converted to log_2_-fold changes by first dividing the average relative quantitation of the treatment group by the control group average and taking the log_2_ of the quotient. A Pearson’s correlation test was calculated using R (version 2.15.2).

### SNP discovery

The samtools package [31] was used for identifying single nucleotide polymorphisms (SNPs) in the same bowtie alignment file used for the differential expression analysis. This file consists of a series of contigs, each containing one assembled transcript and the trimmed sequences from all four cDNA libraries that aligned to that transcript. A custom Perl script pipeline was created to run the mpileup, bcftools, and rmdup commands of the samtools package. To validate a subset of the SNPs identified by samtools, we used a previously published blue crab data set of protein-coding sequences derived from partial exonic regions of five genes [26]. This validation step was done using a custom Perl script to compare the location and alleles of SNPs in regions of the contigs that correspond to the published sequences.

### Availability of supporting data

All sequence data are available on the NCBI GenBank website (BioProject Accession: 283615).

## Additional files

Additional file 1:
**Table of gene ontology (GO) terms for blue crab transcriptome sequences with BLASTX hits in the SwissProt database.** Excel file with tables listing blue crab transcriptome sequences by ID (generated by the Trinity assembler program), SwissProt sequence ID, evidence code, sequence name, and taxon of origin for all of the blue crab sequences that returned as a top BLASTX hit a sequence with GO term annotations that matched any of the following: ‘cold’, ‘defense’, ‘detox’, ‘ecdys’, ‘heat’, ‘hypoxia’, ‘osmo’ or ‘xenobio’. Additional file 2:
**Table of significantly upregulated and downregulated genes and transcripts in gill and hepatopancreas tissues.** Excel file with a table reporting the genes and transcripts that were significantly differentially expressed in each tissue at a false discovery rate (FDR) of 0.05. Genes and transcripts are ranked by log_2_-fold change within each category and when necessary the list is limited to 100.

## Abbreviations

ABI: Applied Biosystems, Inc.
ASV: Alternatively Spliced Variant
BLASTN: Basic Local Alignment Search Tool (nucleotide search)
cDNA: Complementary DNA
CW: Carapace Width
DHOS: Deepwater Horizon Oil Spill
DNA: Deoxyribose Nucleic Acid
FDR: False Discovery Rate
GB: Gigabyte
GO: Gene Ontology
HP: Hewlett Packard
mRNA: Messenger Ribonucleic Acid
NCBI: National Center for Biotechnology Information
qPCR: Quantitative Polymerase Chain Reaction
RAM: Random Access Memory
RNA: Ribonucleic Acid
SD: Standard Deviation
SNP: Single Nucleotide Polymorphism
WAF: Water-accommodated Fraction
